# Plant Density and Nitrogen Supply Affect the Grain-Filling Parameters of Maize Kernels Located in Different Ear Positions

**DOI:** 10.3389/fpls.2019.00180

**Published:** 2019-03-01

**Authors:** Shanshan Wei, Xiangyu Wang, Guanghao Li, Yingying Qin, Dong Jiang, Shuting Dong

**Affiliations:** ^1^College of Agriculture, Nanjing Agricultural University, Nanjing, China; ^2^State Key Laboratory of Crop Biology, College of Agriculture, Shandong Agricultural University, Tai’an, China

**Keywords:** canonical correlation analysis, grain-filling characteristic, kernel position, hormone, logistic curve, maize

## Abstract

Although yield output of maize (*Zea mays* L.) has improved markedly over the last century, procedures for improving the grain-filling process remain elusive. Our aim in this study was to relate grain-filling variation in maize (including kernels in apical and middle positions in the ears) to plant density and nitrogen (N) application rate using a crossed experimental design. We also investigated changes in zeatin riboside (ZR), indole-3-acetic acid (IAA), abscisic acid (ABA), and gibberellic acid (GA) in the kernels during the grain-filling period. Two high-yield maize varieties cultivated extensively in China were field grown under normal (67,500 pl ha^-1^) and high (97,500 pl ha^-1^) densities, and supplied with low, normal and high (0, 180, and 360 kg N ha^-1^) concentrations of N. Kernel weight (KW), the maximum grain-filling rate (G_max_), the average grain-filling rate (G_ave_), and the kernel weight increment achieving G_max_ (W_max_) were all significantly depressed under high density (HD) conditions, but increased N supply partially offset the losses. The apical kernels were more sensitive to density and N application rate than middle kernels. Correlation analysis indicated that plant density and N rate affected KW mainly by influencing the grain-filling rate. Variation in ZR, IAA, and ABA content tracked the variation in KW, but variation in GA content did not. Furthermore, the grain-filling parameters (closely related to TKW) had strong canonical correlation with the content of all hormones across the filling period and ZR content had the strongest relationship. Based on our study, high N supply is beneficial to optimize grain-filling parameters and improve KW of maize kernels under crowded condition.

## Introduction

A gradual increase in plant density has been a vital contributor to maize yield enhancement worldwide (Tokatlidis and Koutroubas, 2004; Antonietta et al., 2014; Testa et al., 2016). However, kernel number per individual plant (KNP) and kernel weight (KW) were decreased due to the exacerbated competition for light and nutrients among individuals under high densities (Tollenaar et al., 2006; Borrás et al., 2007; Boomsma et al., 2009; Al-Naggar et al., 2015). Maize yield is determined by the kernel number per unit area (KNA) and the kernel weight (KW) (Borrás and Otegui, 2001; Mansfield and Mumm, 2014). Once the number of kernels is established, the final KW becomes a main factor determining maize yield (Wang et al., 2012). Hence, improving KW under crowded conditions remains a challenging issue for further enhancements in maize production.

The final weight of maize kernels is generally determined by genotype characteristics (Reddy and Daynard, 1983), which varies greatly with the position on the ear (cob) (Xu et al., 2013), and is also affected by the growth environment (Gambin et al., 2006; Zhou et al., 2017). Sub-optimal conditions related to population density, nutrient status, temperature, drought, and light intensity may restrict kernel set and development (Borrás et al., 2003; Brekke et al., 2011), as well limit the maximum weight achievable (Ouattar et al., 1987; Borras and Gambin, 2010; Shi et al., 2013). For KW, maize grains are usually classified as inferior grains or superior grains, where the former are generally located on the upper part of the ear, and the latter are mostly located on the middle and lower parts of the ear (Chen Y.J. et al., 2013). KW varies with the rate of kernel growth and the duration of grain-filling (Gambin et al., 2007; Sadras and Egli, 2008), which are directly related to grain weight and yield (Borras et al., 2009; Chen T. et al., 2013). During much of the grain-filling period, grain dry weight increases at an essentially linear rate over time. This period of linear increase is known as the effective filling-period duration (EFPD) (Johnson and Tanner, 1972). Borrás and Otegui (2001) reported that KW was closely related to the rate of kernel growth during the EFPD rather than the duration of this period. However, Bolaños (1995) argued that improvements in maize yield were largely explained by the prolongation of the grain-filling period.

The grain-filling process can be accurately modeled with logistic equations, thereby generating a series of important grain-filling parameters, such as maximum grain-filling rate (G_max_), average grain-filling rate (G_ave_) and active grain-filling period (Xu et al., 2013). Besides, the whole grain-filling process could be further divided to three periods according to the shape of the logistic curve, i.e., (i) gradual increase period (GIP), (ii) rapid increase period (RIP), and (iii) slight increase period (SIP) (Wang et al., 2014). Previous work showed the period of maximal grain-filling rate was protracted as fertilizer dosage increased, and the whole process of grain-filling was prolonged (Cao et al., 2008). Xu et al. (2016) indicated the G_max_ and G_ave_ varied with different sowing dates, but the duration of the active grain-filling period did not. Variations of light intensity also affected grain yield by changing the G_max_ (Shi et al., 2013).

Exogenous growth conditions and endogenous plant hormones are both key regulators of kernel development (Lv et al., 2017). Plant hormones play important roles in the development and enrichment of maize, wheat and rice endosperms (Yang et al., 2000; Zhang et al., 2009; Liang et al., 2017). For instance, high zeatin (Z) and zeatin riboside (ZR) content in the initial stage of grain-filling can promote kernel setting and enhance maximum endosperm cell division rate (Morris et al., 1993; Yang et al., 2002). Auxins, gibberellic acid (GA), and abscisic acid (ABA) also regulate grain development (Kende and Zeevaart, 1997; Hansen and Grossmann, 2000; Yang et al., 2006; Zhu et al., 2011). Xu et al. (2013) showed that kernel filling rate was strongly positively correlated with the concentration of Z+ZR, IAA, and ABA in the kernels, but significantly negatively correlated with concentration of GA_3_. Besides, the relative level of hormone in both superior and inferior florets were shown to determine the development of inferior florets (Wang et al., 2006). All early reports have shown that the grain-filling of kernels is controlled by hormones.

Improvements in grain-filling remain a substantial challenge for modern crop production systems with high yield output (Kato et al., 2007). Previous studies regarding grain-filling, especially the relationships between different pollination time and position, plant growth rate at different growth periods, various resource-sink ratio and kernel number set and KW have been established under diverse stress conditions (Zinselmeier et al., 1999; Andrade et al., 2002; Uribelarrea et al., 2002; Gambin et al., 2006). Nevertheless, many studies were concentrated on the early filling stage or the filling rate over the entire filling period. Besides, studies on grain-filling characteristics and KW were mainly focused on variety comparisons, ecological conditions and modifications of single cultivation practices. However, these previous studies showed no evidence on the response of grain-filling characteristics to contrasting stand densities and N availabilities. Information on the relationship between kernel ear position and grain-filling characteristics is also limited. Thus, we examined the grain-filling parameters of maize kernels (including apical and middle position kernels in the ears) under different plant densities and N supply rates using logistic modeling. Besides, the hormone content in grains were determined to analyze their relationships with grain-filling characteristics.

## Materials and Methods

### Trial Site and Conditions

Field evaluations were performed at the Shandong Agricultural University Experimental Farm in Shandong, China during 2014 growing season. This area has a semi-humid, warm temperate continental climate with monsoons. The average monthly maximum temperature, minimum temperature, total solar radiation, and precipitation during the experimental period are shown in Table 1. The soil at the site is neutral sandy loam, and the nutrient status of the top 30 cm before seeding consisted of 11.1 g kg^-1^ organic matter, 0.76 g kg^-1^ available N, 25.4 mg kg^-1^ available phosphate and 105.1 mg kg^-1^ available potassium.

**Table 1:** Monthly average maximum temperature (Tmax, °C) and minimum temperature (Tmin, °C) and monthly total solar radiation (SR, MJ m^-2^) and rainfall (mm) during the growing season (June to September).

Month	Tmax	Tmin	SR	Rainfall
June	30.4	21.2	211.5	34.3
July	33.4	23.0	519.4	44.3
August	31.7	21.3	464.5	28.6
September	27.4	17.8	292.4	94.8
Average/total	30.7	20.8	1487.8	202.0

### Experimental Design

Two high-yield varieties planted widely in local production were selected as the testing materials, i.e., Denghai618 (521 **×** DH392) and Denghai605 (DH351 **×** DH382). The common density was 67,500 pl ha^-1^ and the N application rate ranged from 180 to 360 kg ha^-1^ in this location (Jin et al., 2012; Gao et al., 2017). Maize seeds were planted with hand planters on June 13 and harvested on September 29. Treatments consisted of two densities 67,500 pl ha^-1^ (low density, LD) and 97,500 pl ha^-1^ (high density, HD) and three N levels, 0 kg ha^-1^ (low N, N0), 180 kg ha^-1^ (normal N, N180), and 360 kg ha^-1^ (high N, N360). The experimental treatments were laid out in a split-split plot design, where the N rate was set as the main plot, and variety and density were set as subplot and sub-subplot with three replicates per treatment. The plot size was 12 m long **×** 3 m wide contained five rows and the space between rows was 60 cm. Plants within a row were placed 24.7 and 17.1 cm apart to achieve population density of 67,500 and 97,500 pl ha^-1^, respectively. Phosphorus (P_2_O_5_) and potassium (K_2_O) fertilizer were applied before sowing, at a rate of 90 and 120 kg ha^-1^, respectively. The N fertilizer was in the form of urea and applied as topdressing in two equal portions—one half at the jointing stage, and the other half at the large bell stage. Irrigation, weeds, diseases, and insect pests were controlled adequately during each growing season so that no factors other than density and N supply limiting growth.

In each plot, at least 50 representative plants with robust and uniform growth were labeled and bagged before silking. After the silks were fully exerted, artificial pollination was conducted uniformly on the same day. The shoot bags were replaced after pollination and removed only after pollen stopped shedding. All data were obtained from three rows of plants in the center of the cultivation plots.

### Sampling and Measurements

#### Grain-Filling Traits

Five labeled ears were sampled from each plot to determine grain-filling parameters at 10, 20, 30, 40, and 50 days after pollination (DAP). Kernels from the apical 5–25% files were collected and mixed to provide samples of apical kernels (inferior kernels). Kernels from the 40 to 60% files (counted from the apex to bottom) were collected and mixed to provide middle position grain (superior kernel) samples. We sampled 200 kernels from the five ears and randomly divided them into two equal portions. One portion was frozen in liquid N for 10 min and then stored at -80°C. The second portion was held in an oven for 30 min at 105°C and then dried to constant weight at 80°C.

Grain-filling process was fitted by the logistic equation *W* = *A/* (1+*B*e^-^*^Ct^*). Where *W* refers to the kernel weight, t refers to days after pollination (the day of pollination set as t_0_ = 0), *A* refers to ultimate growth mass, *B* refers to primary parameter and *C* refers to growth rate parameter, respectively. The grain-filling parameters of the tested maize varieties were calculated as follows.

Occurrence time of maximal grain-filling rate (*T*_max_) = ln*B*/*C*;
Kernel weight increment achieving maximum grain-filling rate (*W_max_*) = *A*/2;
Maximum grain-filling rate (*G_max_*) = (*C* **×** *W*_max_) [1-(*W*_max_/*A*)];
Initial filling power (*R*_0_) = *C*;
Active grain-filling period (*P*) = 6/*C.*

The whole process of grain-filling was divided into three periods according to the shape of the logistic curve: (i) gradual increase period, (ii) rapid increase period, and (iii) slight increase period. The first and second order derivatives of the logistic equation were calculated to determine the filling parameters of these three grain-filling periods. The start time of peak grain-filling (t_1_) = (ln B-1.317)/C, the grain weight w_1_ = A/(1+Be^-Ct1^); the end time of peak grain-filling (t_2_) = (ln B+1.317)/C, the grain weight w_2_ = A/(1+Be^-Ct2^); the effective grain-filling period (when the grain weight reached 99% A, t_3_) = (ln B+4.59512)/C, the grain weight was w_3_. The grain-filling parameters of the three grain-filling period were calculated as the following equations.

The gradual increase period (T_1_) = t_1_-t_0_;
The rapid increase period (T_2_) = t_2_-t_1_;
The slight increase period (T_3_) = t_3_-t_2_;
The increment in grain weight during gradual increase period (W_1_) = w_1_-w_0_;
The increment in grain weight during rapid increase period (W_2_) = w_2_-w_1_;
The increment in grain weight during slight increase period (W_3_) = w_3_-w_2_;
The average grain-filling rate during gradual increase period (G_1_) = W_1_/T_1_;
The average grain-filling rate during rapid increase period (G_2_) = W_2_/T_2_;
The average grain-filling rate during slight increase period (G_3_) = W_3_/T_3_.

Besides, the average grain-filling rate during the whole grain-filling period was calculated as:

Average grain-filling rate (G_ave_) = W_3_/t_3_.

#### Hormone Content

The endogenous ABA, IAA, GA, and ZR were extracted with 80% (v/v) methanol and quantified by enzyme-linked immunosorbent assay (ELISA) according to previous studies (Liang et al., 2017). The ELISA kits were provided by the Phytohormones Research Institute, China Agricultural University. The recovery rates for ABA, IAA, GA, and ZR were (90.2 ± 4.1), (86.1 ± 4.5), (79.4 ± 5.3), and (93.4 ± 6.5)%, respectively.

#### Grain Yield, Kernel Weight, and Kernel Number

At physiological maturity, 30 ears from the center three rows of each plot were harvested and air-dried to investigate yield, KNP and 1000-kernel weight (TKW). Grain yield (Mg ha^-1^) was expressed at 15.5% moisture.

### Statistical Analysis

The Curve Expert 1.3 software was used to fit the grain-filling process equation and obtain parameters A, B, and C. Analysis of variance (ANOVA) was performed with SPSS ver. 18.0 (SPSS Institute, Inc.). Duncan’s multiple range test was used to evaluate differences among treatments, and the significance level was set at the 0.05 probability level. Pearson correlation analysis and canonical correlation analysis were also performed. Figures in the article were plotted using SigmaPlot ver. 12.0 and GraphPad Prism ver. 7.

## Results

### Grain Yield and Yield Components

Density, N rate, maize variety and the interactions between them (the density **×** variety interaction term was not included) had significant effects on GY (Table 2). The highest GY for variety DH618 was obtained under a combination of high density (HD) and high N rate (360 kg N ha^-1^, N360). The highest yield of variety DH605 was obtained with a combination of HD and a N rate of 180 kg ha^-1^ (N180). The GY response of DH618 to N (N180, N360, vs. N0) was greater under HD (GY increases of 38.1 and 46.3% with N180 and N360 fertilization, respectively) than at low density (LD) (GY increases of 31.5 and 32.4%, respectively). In DH605, the respective GY increases under HD were smaller: 22.2% (N180) and 22.5% (N360).

**Table 2:** Effects of density and N rate on grain yield (155 g kg^-1^ water content), kernel number and kernel weight.

		Grain yield		Kernel number		1000-Kernel	
Factors		(Mg ha^-^^1^)		(n. ear^-^^1^)		weight (g)	
		DH618	DH605	DH618	DH605	DH618	DH605
LD	N0	10.4e	11.4d	452.6b	536.5b	314.0c	284.4c
	N180	13.7c	13.1b	524.0a	569.8a	331.4b	295.0b
	N360	13.8c	13.2b	544.0a	573.4a	340.7a	298.9a
HD	N0	10.8d	12.0c	348.4c	425.6d	290.4e	269.0d
	N180	15.0b	14.6a	435.8b	479.5c	306.7d	281.8c
	N360	15.8a	14.7a	455.5b	484.1c	313.4c	282.1c
ANOVA							
Density (D)		1141.9^∗∗∗^		384.2^∗∗∗^		857.9^∗∗∗^	
N rate (N)		3285.5^∗∗∗^		87.8^∗∗∗^		282.1^∗∗∗^	
Variety (V)		5.4^∗^		112.1^∗^		2002.9^∗∗∗^	
D × N		108.5^∗∗∗^		1.6^ns^		1.9^ns^	
D × V		0.2^ns^		0.1^ns^		53.2^∗∗∗^	
N × V		262.9^∗∗∗^		9.9^∗∗∗^		21.5^∗∗∗^	
D × N × V		12.5^∗∗∗^		0^ns^		0.5^ns^	

*Different lowercase letters indicate a significant difference (*P* < 0.05) within the same variety among treatments. The numbers in the ANOVA section represent *F*-values. ^*ns*^ Not significant; ^∗^Significant at the 0.05 probability level; ^∗∗^Significant at the 0.01 probability level; ^∗∗∗^Significant at the 0.001 probability level. LD and HD refer to low density and high density, respectively. N0, N180, and N360 represent nitrogen rates of 0, 180, and 360 kg ha^-*1*^,respectively*.

Density and N rate had significant effects on KNP and 1000-kernel weight (TKW), but the density **×** N interaction term was not significant (*P* > 0.05). Increases in stand density were accompanied by decreases in KNP and TKW, but the values of both of these response parameters increased with increasing N rate, especially under HD (KNP values for DH618 increased on average by 27.9 and 17.9% under HD and LD, respectively). The KNP response of DH618 (average 23.0%) exceeded that of DH605 (average 9.9%). The difference in TKW between the two densities as the N rate increased was less marked than the KNP response. The TKW responses of DH618 exceeded those of DH605. For example, in DH618, the TKW value in the N360 **×** HD treatment increased by 7.9% in comparison with the N0 treatment, whereas the TKW proportional response to the same treatment was 4.8% in DH605.

### Grain Weight Dynamic

**Figure 1:**
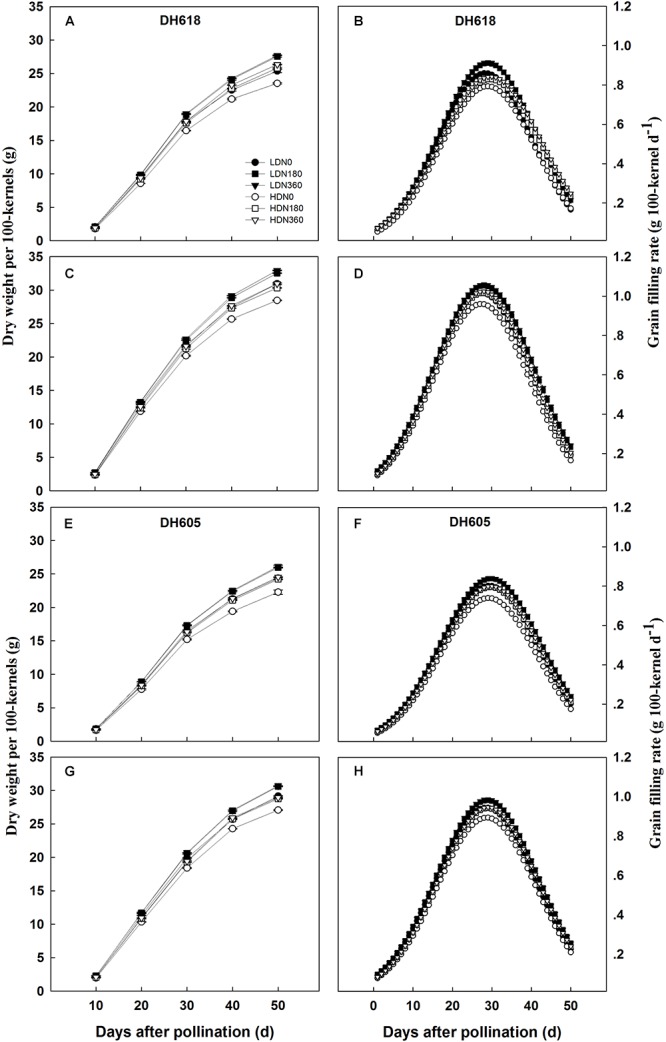
Effects of density and nitrogen application rate on the grain dry weight and grain-filling rate of **(A,B,E,F)** upper kernels and **(C,D,G,H)** middle kernels **(A–D)** in Denghai618 and **(E–H)** Denghai605 during the 2014 growing season. LD and HD refer to low density and high density, respectively. N0, N180, and N360 represent nitrogen rates of 0, 180, and 360 kg ha^-1^, respectively.

Density, N rate and variety significantly affected KW in both the apical and middle ear positions (data not shown). The density **×** N rate interaction term was significant after 10 DAP for middle position kernels, but not for the apical grains. The KW of middle position kernels was higher than that of apical kernels over the whole grain-filling period. In each of the grain-filling periods, increased density reduced KW, while increased N application rates improved KW values (Figure 1), as expected. The responses of kernels in different ear positions were always different. Apical kernels responded more strongly to density and N supply than middle position kernels. Thus, in variety DH618, the KW values of apical and middle position kernels under HD were reduced on average by 8.6 and 5.9%, respectively, in comparison with the LD treatment. The respective average KW values in the high N treatment (N360) were 7.7 and 6.3% above the values in the N0 treatment. The response to N (N180, N360, vs. N0) was greater at HD. For example, under HD, the KW values of middle position kernels of DH618 at 30 DAP were 5.3% (N180) and 7.0% (N360) higher than those in the N0 treatment, whereas the respective increases under LD were 3.4% (N180) and 4.6% (N360).

### Grain-Filling Characteristic Parameters

**Figure 2:**
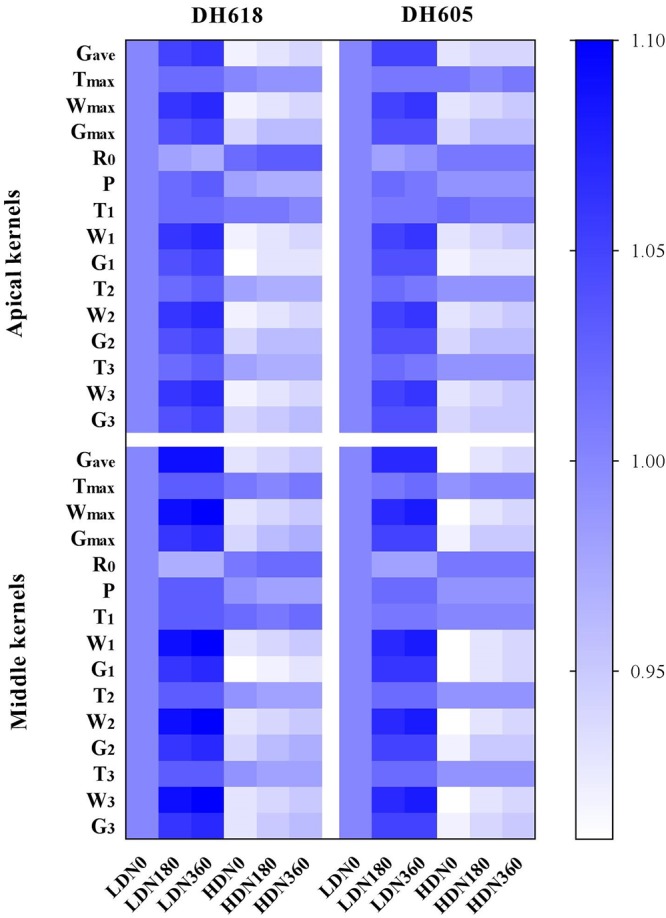
The variations of grain-filling characteristic parameters under different density and N application rate combinations. The results are the ratios of grain-filling characteristic parameters values under different D × N combinations to LDN0 for each variety (i.e., ratios of LDN180/LDN0, HDN180/LDN0, et al.), where LD and HD refer to low density and high density, respectively. N0, N180, and N360 represent nitrogen rates of 0, 180, and 360 kg ha^-1^, respectively. G_ave_, average grain-filling rate; T_max_, the day reaching the maximum grain-filling rate; W_max_, kernel weight increment achieving maximum grain-filling rate; G_max_, maximum grain-filling rate; R_0_, initial grain-filling potential; P, active grain-filling period; T_1_, grain-filling duration of gradual increase period; W_1_, increased grain weight of gradual increase period; G_1_, mean grain-filling rate of gradual increase period; T_2_, grain-filling duration of rapid increase period; W_2_, increased grain weight of rapid increase period; G_2_, mean grain-filling rate of rapid increase period; T_3_, grain-filling duration of slight increase period; W_3_, increased grain weight of slight increase period; G_3_, mean grain-filling rate of slight increase period.

We divided the grain-filling parameter values under different D **×** N combinations by the value for the LDN0 treatment. These ratios are listed in Supplementary Table S1 and are presented in Figure 2. At 10 DAP, the different density and N rate combinations showed no significant effects. However, after 20 DAP, the differences in grain-filling rates among treatments became obvious. Progress in grain-filling was well-simulated by the Richards model: all correlation coefficients were > 0.99 (Table 3). The maximum grain-filling rate (G_max_), the kernel weight increment achieving G_max_ (W_max_), average grain-filling rate (G_ave_) and the active grain-filling period (P) all decreased with increased density, but increased with the N supply rate. The initial grain-filling potential (R_0_) and the day on which the maximum grain-filling rate (T_max_) reached responded differently. Compared with the values for superior kernels, the G_max_, G_ave_, and *P*-values of inferior kernels were depressed. However, apical kernels responded more strongly to N supply. G_max_ values in treatment N360 (DH618) were elevated by 8.6 and 5.6% in apical and middle position kernels, respectively, above the values in the N0 treatment. The grain-filling rate was higher in DH618 than in DH605, but the active grain-filling period of DH618 was shorter.

**Table 3:** Effects of density and N rate on grain-filling characteristics parameters of maize kernels at upper and middle position.

Kernel position	Variety	Density	N rate	R^2^	A	B	C	T_max_	W_max_	G_max_	G_ave_	R_0_	P
								(d)	(g 100-kernels)	(g 100-kernels d^-1^)			(d)
Upper	DH618	LD	N0	0.998	25.28	38.38	0.15	24.06	12.64	0.96	0.51	0.15	39.58
			N180	0.998	27.55	38.21	0.15	24.69	13.77	1.02	0.55	0.15	40.67
			N360	0.998	27.71	38.21	0.15	24.69	13.85	1.02	0.55	0.15	40.66
		HD	N0	0.999	23.58	41.50	0.15	24.30	11.79	0.90	0.47	0.15	39.14
			N180	0.998	25.77	41.82	0.15	24.70	12.89	0.97	0.52	0.15	39.69
			N360	0.998	26.39	41.42	0.15	24.85	13.19	0.99	0.53	0.15	40.04
	DH605	LD	N0	0.998	24.43	39.03	0.15	24.99	12.22	0.90	0.49	0.15	40.92
			N180	0.998	26.09	38.29	0.14	25.36	13.05	0.94	0.52	0.14	41.74
			N360	0.998	26.27	38.17	0.14	25.42	13.14	0.94	0.52	0.14	41.88
		HD	N0	0.998	22.25	39.99	0.15	24.85	11.12	0.83	0.45	0.15	40.43
			N180	0.998	24.32	39.72	0.15	25.25	12.16	0.89	0.48	0.15	41.15
			N360	0.998	24.69	39.34	0.14	25.38	12.35	0.89	0.49	0.14	41.47
Middle	DH618	LD	N0	0.997	30.71	31.21	0.15	23.36	15.36	1.13	0.62	0.15	40.73
			N180	0.997	32.48	31.15	0.14	23.79	16.24	1.17	0.65	0.14	41.52
			N360	0.997	32.92	30.95	0.14	23.90	16.46	1.18	0.66	0.14	41.78
		HD	N0	0.997	28.32	33.49	0.15	23.28	14.16	1.07	0.57	0.15	39.79
			N180	0.997	30.27	33.96	0.15	23.60	15.14	1.13	0.61	0.15	40.17
			N360	0.997	30.88	33.13	0.15	23.69	15.44	1.14	0.62	0.15	40.61
	DH605	LD	N0	0.997	29.16	33.61	0.14	24.31	14.58	1.05	0.58	0.14	41.50
			N180	0.997	30.75	33.24	0.14	24.62	15.37	1.09	0.61	0.14	42.15
			N360	0.997	30.83	33.63	0.14	24.61	15.42	1.10	0.61	0.14	42.00
		HD	N0	0.998	27.24	35.66	0.15	24.47	13.62	0.99	0.54	0.15	41.09
			N180	0.998	29.05	35.33	0.14	24.73	14.52	1.05	0.58	0.14	41.62
			N360	0.998	29.22	35.45	0.14	24.75	14.61	1.05	0.58	0.14	41.62

*A, B, C represent equation coefficient; T_*max*_, the day reaching the maximum grain-filling rate; W_*max*_, kernel weight increment achieving maximum grain-filling rate; G_*max*_, maximum grain-filling rate; G_*ave*_, average grain-filling rate; R_*0*_, initial grain-filling potential; P, active grain-filling period*.

Density increases prolonged the duration of the GIP in most cases, but shortened the durations of two other periods on the logistic curve. The GIP, RIP and SIP durations were all prolonged in both inferior and superior kernels of the two varieties by increasing the N supply rate (Table 4). The increases in KW of inferior kernels through all the three filling periods were smaller compared with the increases in superior kernels. The duration of the apical kernel RIP and SIP were shorter, but the duration of the GIP was longer in comparison with middle position kernels. In addition, the duration of the DH618 GIP was shorter than the DH605 GIP, but the grain-filling rate and the increases in grain weight during the GIP were higher in DH618.

**Table 4:** Effects of density and N rate on characteristics parameters of the three grain-filling phases of maize kernels at upper and middle position.

Kernel position	Variety	Density	N rate	Gradual increase period			Rapid increase period			Slight increase period		
				T_1_	W_1_	G_1_	T_2_	W_2_	G_2_	T_3_	W_3_	G_3_
Upper	DH618	LD	N0	15.37	5.34	0.35	17.37	14.60	0.84	21.62	5.09	0.46
			N180	15.77	5.82	0.37	17.85	15.91	0.89	22.22	5.55	0.49
			N360	15.76	5.86	0.37	17.85	16.00	0.90	22.22	5.58	0.49
		HD	N0	15.71	4.98	0.32	17.18	13.61	0.79	21.38	4.75	0.43
			N180	15.99	5.45	0.34	17.43	14.88	0.85	21.69	5.19	0.46
			N360	16.06	5.58	0.35	17.58	15.24	0.87	21.88	5.31	0.47
	DH605	LD	N0	16.01	5.16	0.32	17.97	14.11	0.79	22.36	4.92	0.43
			N180	16.20	5.51	0.34	18.32	15.07	0.82	22.80	5.25	0.45
			N360	16.23	5.55	0.34	18.38	15.17	0.83	22.88	5.29	0.45
		HD	N0	15.98	4.70	0.29	17.75	12.85	0.72	22.09	4.48	0.39
			N180	16.22	5.14	0.32	18.07	14.04	0.78	22.48	4.90	0.42
			N360	16.28	5.22	0.32	18.20	14.26	0.78	22.66	4.97	0.43
Middle	DH618	LD	N0	14.42	6.49	0.45	17.88	17.73	0.99	22.25	6.18	0.56
			N180	14.68	6.86	0.47	18.23	18.75	1.03	22.68	6.54	0.58
			N360	14.73	6.96	0.47	18.34	19.01	1.04	22.83	6.63	0.58
		HD	N0	14.55	5.98	0.41	17.47	16.35	0.94	21.74	5.70	0.52
			N180	14.78	6.40	0.43	17.63	17.48	0.99	21.95	6.09	0.55
			N360	14.78	6.53	0.44	17.83	17.83	1.00	22.19	6.22	0.56
	DH605	LD	N0	15.20	6.16	0.41	18.22	16.83	0.92	22.67	5.87	0.51
			N180	15.36	6.50	0.42	18.51	17.75	0.96	23.03	6.19	0.53
			N360	15.39	6.52	0.42	18.44	17.80	0.97	22.95	6.21	0.54
		HD	N0	15.46	5.76	0.37	18.04	15.73	0.87	22.45	5.48	0.48
			N180	15.59	6.14	0.39	18.27	16.77	0.92	22.74	5.85	0.51
			N360	15.61	6.17	0.40	18.27	16.87	0.92	22.74	5.88	0.51

*T_*1*_, grain-filling duration of gradual increase period; W_*1*_, increased grain weight of gradual increase period; G_*1*_, mean grain-filling rate of gradual increase period; T_*2*_, grain-filling duration of rapid increase period; W_*2*_, increased grain weight of rapid increase period; G_*2*_, mean grain-filling rate of rapid increase period; T_*3*_, grain-filling duration of slight increase period; W_*3*_, increased grain weight of slight increase period; G_*3*_, mean grain-filling rate of slight increase period*.

### Variations in Hormone Content

**Figure 3:**
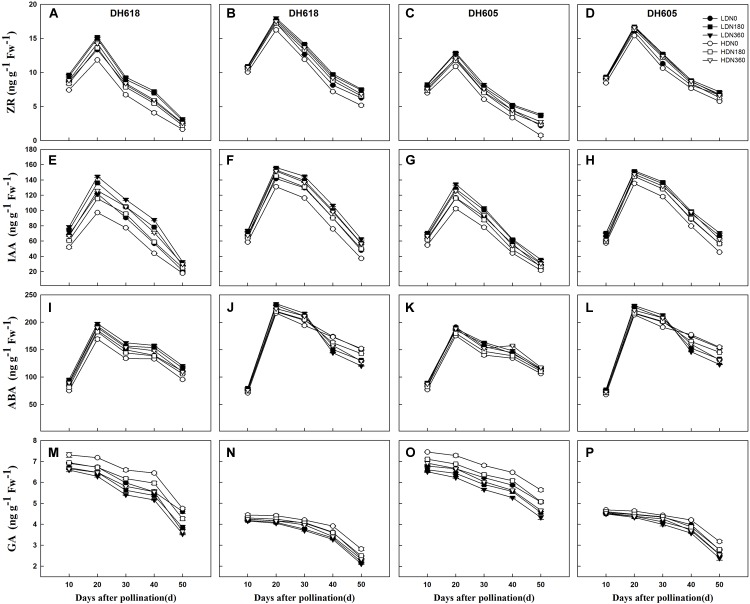
Effects of density and nitrogen application rate on the content of zeatin riboside (ZR), indole-3-acetic acid (IAA), abscisic acid (ABA), and gibberellic acid (GA) in **(A,C,E,G,I,K,M,O)** upper kernels and **(B,D,F,H,J,L,N,P)** middle kernels of the two varieties during 2014 growing season. LD and HD refer to low density and high density, respectively. N0, N180, and N360 represent nitrogen rates of 0, 180, and 360 kg ha^-1^, respectively.

During the grain-filling process, the overall trends in IAA, ZR, and ABA content in the grains showed unimodal curves. The GA content declined continually in all treatments (Figure 3). Plant density and N rate both had significant effects on hormonal content during the whole filling period. The density **×** N rate interaction was significant for most hormone content (Supplementary Table S2). Density and N rate did not alter the general trends of any of the hormones over time. At each grain-filling stage, IAA and ZR content were higher in middle position kernels than in the apical kernels, but the GA content was higher in the apical kernels. Before 30 DAP, the concentration of ABA was much lower in the apical kernels than in the middle position grains. The hormone content in apical kernels responded more strongly to density and N supply than in middle position kernels. For example, the Z+ZR content at 20 DAP was reduced by average 8.8 and 3.7% (HD vs. LD), but were increased by average 17.3 and 5.9% (N360 vs. N0) for inferior and superior kernels, respectively. The response to N (N180, N360, vs. N0) was also elevated in the HD treatment. Thus, the Z+ZR content at 20 DAP (middle position kernels of DH618) was increased by 2.8% (N180) and 3.5% (N360) under LD, and by 6.2% (N180) and 7.7% (N360) under HD.

### Correlation Between 1000-Grain Weight and Grain-Filling Parameters

We examined the relationships between grain-filling parameters in different ear positions and the TKW (Table 5). Correlation analysis showed that G_ave_, W_max_, G_max_, the grain-filling rate and KW increased during the GIP, RIP, and SIP were significantly positively correlated with TKW at physiological maturity in kernels at both ear positions. We also found a significant negative relationship between KW and the duration of the GIP in middle position kernels.

**Table 5:** Pearson correlation of 1000-kernel weight (TKW) and filling parameters of maize kernels at different ear positions.

	Kernel
	position	G_ave_	T_max_	W_max_	G_max_	R_0_	D	T_1_	W_1_	G_1_	T_2_	W_2_	G_2_	T_3_	W_3_	G_3_
TKW	Upper	0.905^∗∗^	-0.402	0.886^∗∗^	0.967^∗∗^	0.244	-0.202	-0.501	0.888^∗∗^	0.963^∗∗^	-0.205	0.887^∗∗^	0.964^∗∗^	-0.201	0.889^∗∗^	0.965^∗∗^
	Middle	0.955^∗∗^	-0.544	0.934^∗∗^	0.977^∗∗^	-0.08	0.021	-0.700^∗^	0.933^∗∗^	0.979^∗∗^	0.02	0.934^∗∗^	0.979^∗∗^	0.021	0.932^∗∗^	0.979^∗∗^

*TKW, 1000- kernel weight; G_*ave*_, average grain-filling rate; T_*max*_, the day reaching the maximum grain-filling rate; W_*max*_, kernel weight increment achieving maximum grain-filling rate; G_*max*_, maximum grain-filling rate; R_*0*_, initial grain-filling potential; P, active grain-filling period; T_*1*_, grain-filling duration of gradual increase period; W_*1*_, increased grain weight of gradual increase period; G_*1*_, mean grain-filling rate of gradual increase period; T_*2*_, grain-filling duration of rapid increase period; W_*2*_, increased grain weight of rapid increase period; G_*2*_, mean grain-filling rate of rapid increase period; T_*3*_, grain-filling duration of slight increase period; W_**3**_, increased grain weight of slight increase period; G_*3*_, mean grain-filling rate of slight increase period. ^∗^Significant at the 0.05 probability level, ^∗∗^Significant at the 0.01 probability level.*

### Correlation Between Grain-Filling Parameters and Hormone Content

Canonical correlation analysis was used to examine the relationships between hormone content (*X*) and grain-filling parameters (*Y*). Hormone content at 10, 20, 30, 40, and 50 DAP were set as variables *x*_1_, *x*_2_, *x*_3_, *x*_4_, and *x*_5_, respectively. G_ave_, W_max_, G_max_, G_1_, G_2_, and G_3_ were set as *y*_1_, *y*_2_, *y*_3_, *y*_4_, *y*_5_, and *y*_6_, respectively. As shown in Table 6, the first canonical correlation coefficients were all statistically significant, indicating that the correlations between the grain-filling parameters and hormone content can be described by the first pair of typical variables (except the ABA content of superior kernels). The linear combinations of the first pair of canonical correlation models are shown in Table 7. From the coefficients for each variable, we were able to show that the grain-filling parameters of apical kernels were mainly determined by the content of ZR at 40 DAP, IAA at 20 DAP, ABA at 10 DAP and GA at 40 DAP, whereas content of ZR at 50 DAP, IAA and ABA at 20 DAP, and GA at 10 DAP were the main factors determining the grain-filling parameters of the middle position kernels. Most information of *X* and *Y* were concentrated in the first pair of canonical variables (*L*1 and *M*1, Table 8). Among the hormones examined in our study, ZR had the strongest correlation with grain-filling parameters of both inferior and superior kernels (86.0 and 79.6% of the ZR variation may affect variation in grain-filling parameters in inferior and superior kernels, respectively; 90.2 and 91.9% of the variation in grain-filling parameters were related to ZR content changes in the inferior and superior kernels, respectively).

**Table 6:** Canonical correlation coefficient between grain filling parameters and hormone content and the significance testing.

Hormone	Number	Upper kernels			Middle kernels		
		Canonical correlation coefficient	χ^2^ Value	*P*-value	Canonical correlation coefficient	χ^2^ Value	*P*-value
ZR	1	1.000	59.378	0.001	0.999	68.630	0.000
	2	0.983	24.140	0.236	0.990	35.295	0.019
	3	0.692	7.275	0.839	0.949	15.836	0.199
	4	0.644	4.018	0.674	0.669	4.299	0.636
	5	0.485	1.342	0.511	0.483	1.330	0.514
IAA	1	0.998	52.774	0.006	1.000	81.803	0.000
	2	0.976	24.639	0.216	0.988	36.438	0.014
	3	0.872	9.444	0.665	0.969	17.829	0.121
	4	0.601	2.301	0.890	0.640	3.793	0.705
	5	0.112	0.063	0.969	0.455	1.162	0.559
ABA	1	0.991	33.033	0.321	0.999	64.411	0.000
	2	0.817	13.053	0.875	0.974	32.123	0.042
	3	0.809	7.545	0.820	0.962	17.290	0.139
	4	0.483	2.224	0.898	0.756	4.364	0.628
	5	0.406	0.899	0.638	0.159	0.129	0.938
GA	1	0.998	64.348	0.000	1.000	70.779	0.000
	2	0.987	35.699	0.017	0.979	31.335	0.051
	3	0.968	17.409	0.135	0.908	15.433	0.219
	4	0.586	3.558	0.736	0.769	6.719	0.348
	5	0.502	1.451	0.484	0.602	2.252	0.324

**Table 7:** Standardized analytical model of canonical variable.

Kernel
position	Hormone	Canonical variable
Upper	ZR	*L*1 = -0.541 *x*_*1*_-0.66 *x*_*2*_-0.52 *x*_*3*_+0.891 *x*_*4*_-0.254 *x*_*5*_
	IAA	*L*1 = 3.704 *x*_*1*_-4.619 *x*_*2*_+0.365 *x*_*3*_-0.72 *x*_*4*_+0.433 *x*_*5*_
	ABA	*L*1 = -1.407 *x*_*1*_-0.017 *x*_*2*_-0.067 *x*_*3*_-0.231 *x*_*4*_+0.876 *x*_*5*_
	GA	*L*1 = 0.198 *x*_*1*_-0.325 *x*_*2*_+0.687 *x*_*3*_-0.933 *x*_*4*_-0.722 *x*_*5*_
Middle	ZR	*L*1 = 0.394 *x*_*1*_-0.054 *x*_*2*_+0.5 *x*_*3*_-0.522 *x*_*4*_+0.859 *x*_*5*_
	IAA	*L*1 = 0.216 *x*_*1*_-1.162 *x*_*2*_+0.527 *x*_*3*_-0.483 *x*_*4*_-0.051 *x*_*5*_
	ABA	*L*1 = -0.295 *x*_*1*_-1.537 *x*_*2*_+0.054 *x*_*3*_-0.089 *x*_*4*_-0.798 *x*_*5*_
	GA	*L*1 = 3.122 *x*_*1*_-3.066 *x*_*2*_-0.43 *x*_*3*_-0.254 *x*_*4*_+1.7 *x*_*5*_

**Table 8:** The proportion of variable variation can be explained by canonical variable (redundancy analysis).

Kernel		Original
position	Hormone	variables	*L*1	*L*2	*L*3	*L*4	*L*5	*L* (*L*1∼*L*5)	*M*1	*M*2	*M*3	*M*4	*M*5	*M* (*M*1∼*M*5)
Upper	ZR	*X*	0.861	0.030	0.006	0.071	0.031	0.999	0.860	0.029	0.003	0.030	0.007	0.929
		*Y*	0.902	0.047	0.014	0.019	0.013	0.995	0.901	0.046	0.007	0.008	0.003	0.965
	IAA	*X*	0.772	0.071	0.075	0.033	0.048	0.999	0.769	0.068	0.057	0.012	0.001	0.907
		*Y*	0.679	0.166	0.080	0.062	0.005	0.992	0.677	0.158	0.061	0.022	0.000	0.918
	ABA	*X*	0.614	0.191	0.077	0.051	0.067	1.000	0.603	0.127	0.050	0.012	0.011	0.803
		*Y*	0.740	0.016	0.050	0.100	0.001	0.907	0.727	0.011	0.033	0.023	0.000	0.794
	GA	*X*	0.861	0.029	0.009	0.084	0.017	1.000	0.858	0.028	0.008	0.029	0.004	0.927
		*Y*	0.806	0.066	0.015	0.013	0.039	0.939	0.803	0.064	0.014	0.005	0.010	0.896
Middle	ZR	*X*	0.797	0.155	0.030	0.004	0.014	1.000	0.796	0.152	0.027	0.002	0.003	0.980
		*Y*	0.919	0.053	0.007	0.010	0.001	0.990	0.919	0.053	0.007	0.010	0.001	0.990
	IAA	*X*	0.814	0.096	0.072	0.009	0.009	1.000	0.814	0.094	0.067	0.004	0.002	0.981
		*Y*	0.328	0.632	0.022	0.013	0.003	0.998	0.328	0.616	0.021	0.005	0.001	0.971
	ABA	*X*	0.696	0.209	0.051	0.028	0.016	1.000	0.694	0.198	0.047	0.016	0.000	0.955
		*Y*	0.777	0.013	0.074	0.087	0.046	0.997	0.776	0.013	0.068	0.050	0.001	0.908
	GA	*X*	0.774	0.136	0.067	0.011	0.012	1.000	0.774	0.131	0.055	0.006	0.004	0.970
		*Y*	0.801	0.156	0.019	0.011	0.012	0.999	0.801	0.149	0.016	0.007	0.004	0.977

## Discussion

Although maize GY is determined mainly by KNA, variations in KW are also influential (Zhou et al., 2016). Grain-filling is a critical physiological process that determines the KW and yield of corn kernels. Poor grain-filling limits the potential GY (Yang and Zhang, 2010) and is among the major scientific and practical issues that should be addressed to improve agricultural production. Appropriate plant densities and N fertilization rates are important elements of efficient management that have contributed to maize yield improvement (Wei et al., 2017). We showed in this study that density, N rate and their interaction affected KW during the whole filling period, while the impact of density tended to be stronger. The variation in KW between different floret positions of the maize ear was substantial and mostly related to changes in the grain-filling rate. Furthermore, the low KW values in apical kernels were exacerbated under HD, though the yield potential under HD was nevertheless 9.8% higher than that under LD. Taken together with the strong response of apical kernels to N rate (especially under HD), we concluded that apical grains were more sensitive to environmental factors than middle grains. Hence, promoting grain-filling of grains at apical position will be key for improving the KW of maize.

The KW is determined by the grain-filling rate, the effective filling duration, or both (Zhou et al., 2017). In our study, G_ave_, G_max_ and the filling rate during GIP, RIP, and SIP were all positively correlated with KW, but we found no obvious correlation between KW and the active grain-filling period in neither superior kernels nor inferior kernels (Table 6). Thus, changes in the filling rate were mainly a result of variation in KW under different density and N rate combinations. Li et al. (2000) showed that difference in KW within the same maize variety was determined by the grain-filling rate, whereas KW variation among varieties was determined by grain-filling duration. We attributed KW differences between the two maize varieties that we tested to differences in filling rate, rather than filling duration. Unlike apical kernels, the duration of the GIP in middle position kernels was strongly negatively correlated to KW. A mechanism for shortening the duration of the GIP whilst maintaining the filling rate could prolong the duration of the subsequent RIP relative to the SIP. This procedure could increase KW.

The processes of cell division, differentiation, enlargement, and enrichment of inclusions during the development of maize kernels are controlled by a range of hormones operating during different filling periods (Yang et al., 2001; Artlip et al., 2010). In the current study, density and N level did not change the regulation period of the hormones in grains, however, hormone content varied significantly through kernel development. HD decreased the highest values, and accelerated reductions in ZR and IAA content, whereas increased N application offset these declines, especially under HD. The strong canonical correlation between grain-filling parameters and ZR content indicated that density and N rate combinations affected grain-filling progress largely by adjusting the content of ZR, which regulated the rates of endosperm cell division and grain filling (Ahmad et al., 2018). Additionally, we also showed that the ZR and IAA content of apical kernels were significantly lower than the content in middle position kernels. Hence, we attributed the lower KW in the apical kernels to their lower ZR and IAA content, these low hormone levels constrained the proliferation of endosperm cells (Wang et al., 2006), reduced the sink capacity, and ultimately decreased KW. ZR and IAA are considered to be the main regulators of endosperm cell development, whereas ABA likely has a vital regulatory role in the process of photosynthate transport and accumulation in the grain (Hansen and Grossmann, 2000; Travaglia et al., 2007; Qin et al., 2013). We found that the ABA concentration in the kernels was reduced by plant crowding, but enhanced by increased N availability, in agreement with the findings of Gawronska et al. (2003). Furthermore, we determined that about 75% of the variation in grain-filling parameters was related to variation in ABA content in both apical and middle position kernels. The ABA content during the early filling period (10–20 DAP) was most strongly positively correlated with the grain-filling parameters, as shown in previous studies on maize, wheat, and rice (Xu et al., 2007, 2013; Zhang et al., 2009; Lv et al., 2017). The combination of elevated ABA concentration after 30 DAP and the low KW in the HD **×** N0 treatment also suggested that maintaining a high level of ABA during the late grain-filling stage may negatively impact the progress of grain-filling.

Unlike the other hormones, the GA levels in both apical and middle position kernels were highest at 10 DAP, when the embryo was enlarging rapidly. In constract to the ZR content, GA content increased when density was increased, but declined with increased N application. The concentration of GA in the apical kernels exceeded that in the middle position kernels. Earlier studies have indicated that GA in the kernels played an important role in the rapid amplification of endosperm cells. However, we found that the GA content in the HD **×** N0 was maintained at a high level over the whole filling period, suggesting that elevated GA content adversely affected the improvement of KW. This outcome is congruent with earlier work (Xu et al., 2013), showing that GA can enhance the activities of α-amylase and other hydrolytic enzyme that promote the decomposition of starch (Yang et al., 2001). Thus, changes in ZR, IAA, ABA, and GA content in grains were all significantly correlated with the grain-filling parameters. Further work is needed to explore the mechanisms controlling (i) hormone content variation under different density **×** N rate combinations, and (ii) hormone distribution within maize ears over the whole grain-filling period.

In short, variation in grain-filling rate was the main factor affecting TKW under different density **×** N rate combinations. The apical kernels were more sensitive to density and N application rate than middle position kernels. High N input weakened the reduction in KW, G_max_, G_ave_, W_max_, RIP, and SIP durations under high density. The grain-filling parameters (closely related to TKW) had strong canonical correlations with the content of all hormones across the filling period, and ZR content had the strongest relationship. Therefore, we conclude from our results that high N supply is beneficial to optimize grain-filling parameters and improve KW of maize kernels under crowded condition.

## Author Contributions

SW designed the work, carried out all experiments and data analysis and drafted the manuscript. XW performed field experiments and sampling and helped drafting the work. SD conceived the study and planned the experiments. GL and YQ helped draft the manuscript. DJ helped draft and revise the manuscript. All authors read and approved the final manuscript.

## Conflict of Interest Statement

The authors declare that the research was conducted in the absence of any commercial or financial relationships that could be construed as a potential conflict of interest.

## Supplementary Material

TABLE S1 The data used in Figure 2.

TABLE S2 Analysis of variance of hormone contents in maize grains under different density and N rate conditions.

## Abbreviations

G_1_: mean grain-filling rate of gradual increase period
G_2_: mean grain-filling rate of rapid increase period
G_3_: mean grain-filling rate of slight increase period
G_ave_: average grain-filling rate
G_max_: maximum grain-filling rate
KW: kernel weight
P: active grain-filling period
R_0_: initial grain-filling potential
T_1_: grain-filling duration of gradual increase period
T_2_: grain-filling duration of rapid increase period
T_3_: grain-filling duration of slight increase period
TKW: 1000-kernel weight
T_max_: the day reaching the maximum grain-filling rate
W_1_: increased grain weight of gradual increase period
W_2_: increased grain weight of rapid increase period
W_3_: increased grain weight of slight increase period
W_max_: kernel weight increment achieving maximum grain-filling rate.
