# Supplementary material for: Plant Density and Nitrogen Supply Affect the Grain-Filling Parameters of Maize Kernels Located in Different Ear Positions
Source: Front Plant Sci. 2019 Mar 1;10:180. doi: 10.3389/fpls.2019.00180 (PMC6405445; doi:10.3389/fpls.2019.00180)
Supplement: TABLE S2 — Analysis of variance of hormone contents in maize grains under different density and N rate conditions. [file Table_2.docx]

Supplementary Material

**Plant density and nitrogen supply affect the grain-filling parameters of maize kernels located in different ear positions**

**Shanshan Wei^1, 2^, Xiangyu Wang^2^, Guanghao Li^2^, Yingying Qin^2^, Dong Jiang^1,*^, Shuting Dong^2,*^**

***Correspondence:** Dong Jiang: [jiangd@njau.edu.cn](mailto:jiangd@njau.edu.cn); Shuting Dong: [stdong@sdau.edu.cn](mailto:stdong@sdau.edu.cn).

**Supplementary Table 2** Analysis of variance of hormone contents in maize grains under different density and N rate conditions.

| Variety | Hormone | Factors | Medium kernels | | | | | Upper kernels | | | | |
| --- | --- | --- | --- | --- | --- | --- | --- | --- | --- | --- | --- | --- |
|  |  |  | 10 DAP | 20 DAP | 30 DAP | 40 DAP | 50 DAP | 10 DAP | 20 DAP | 30 DAP | 40 DAP | 50 DAP |
| Denghai618 | GA | ANOVA |  |  |  |  |  |  |  |  |  |  |
|  |  | Density(D) | 44.9*** | 94.9*** | 107.3*** | 119.4*** | 192.2*** | 54.6*** | 132.8*** | 357.6*** | 467.6*** | 25.1*** |
|  |  | N rate(N) | 17.8*** | 34.5*** | 69.7*** | 142*** | 106.7*** | 79.9*** | 171.5*** | 200.7*** | 165.2*** | 195.5*** |
|  |  | D×N | 2.1ns | 5.5* | 5.7* | 4.4* | 5.6* | 9.8** | 9.9** | 4.8* | 26.5*** | 4.3* |
|  | ZR | ANOVA |  |  |  |  |  |  |  |  |  |  |
|  |  | Density(D) | 130.8*** | 66.7*** | 598.3*** | 640.1*** | 471.7*** | 717.8*** | 1038.7*** | 336.2*** | 973.5*** | 444.4*** |
|  |  | N rate(N) | 171.6*** | 55.7*** | 1606.1*** | 1430.9*** | 502.9*** | 329.6*** | 1126.7*** | 169.1*** | 456.1*** | 191*** |
|  |  | D×N | 5.6* | 4.8* | 4.8* | 10.9** | 6.8** | 9.7** | 17.3*** | 6.1* | 5.4* | 4.7* |
|  | ABA | ANOVA |  |  |  |  |  |  |  |  |  |  |
|  |  | Density(D) | 379.4*** | 171*** | 134*** | 154.8*** | 116.1*** | 177.9*** | 137.5*** | 213.4*** | 238.1*** | 692.5*** |
|  |  | N rate(N) | 94.1*** | 141.5*** | 216.1*** | 410.9*** | 497.6*** | 107.3*** | 83.5*** | 160.6*** | 205.9*** | 225.9*** |
|  |  | D×N | 5* | 19.3*** | 5.5* | 46.7*** | 22.5*** | 5.5* | 4.3* | 11.1** | 14.1** | 11.2** |
|  | IAA | ANOVA |  |  |  |  |  |  |  |  |  |  |
|  |  | Density(D) | 428.4*** | 356.8*** | 337.9*** | 687.5*** | 265.7*** | 789*** | 2137.9*** | 402.3*** | 1341.9*** | 37.5*** |
|  |  | N rate(N) | 143.5*** | 661.3*** | 381.2*** | 758.9*** | 418.7*** | 209.9*** | 1054.7*** | 811.7*** | 1452.6*** | 197.6*** |
|  |  | D×N | 4.4* | 18*** | 10.7** | 20.7*** | 8.1** | 6.3* | 15.8*** | 6.6* | 16.2*** | 0.1ns |
| Denghai605 | GA | ANOVA |  |  |  |  |  |  |  |  |  |  |
|  |  | Density(D) | 85.9*** | 43.2*** | 73.8*** | 92*** | 108.7*** | 436.3*** | 451*** | 557.6*** | 368.3*** | 229.7*** |
|  |  | N rate(N) | 42.4*** | 49.9*** | 41.3*** | 274.5*** | 140.6*** | 86.9*** | 164.3*** | 376.6*** | 267.6*** | 249.2*** |
|  |  | D×N | 4.8* | 4.5* | 5.6* | 5.3* | 6.9** | 8.8** | 7.7** | 8.8** | 9.4** | 4.9* |
|  | ZR | ANOVA |  |  |  |  |  |  |  |  |  |  |
|  |  | Density(D) | 90.3*** | 80.3*** | 181.6*** | 44.9*** | 198.7*** | 678.9*** | 632.9*** | 453.7*** | 785.8*** | 306.2*** |
|  |  | N rate(N) | 107.4*** | 220.6*** | 815.6*** | 142.2*** | 73.1*** | 85.9*** | 277.8*** | 419.6*** | 242.6*** | 253.7*** |
|  |  | D×N | 12.1** | 8.2** | 7.5** | 4.3* | 8.6** | 5.3* | 4.8* | 10.6** | 7.5** | 1.9ns |
|  | ABA | ANOVA |  |  |  |  |  |  |  |  |  |  |
|  |  | Density(D) | 247.5*** | 144.1*** | 174*** | 895.3*** | 153*** | 148.4*** | 385.1*** | 42*** | 6.6* | 12.6** |
|  |  | N rate(N) | 64.9*** | 123.7*** | 263.5*** | 1779.8*** | 661.6*** | 144.1*** | 77.7*** | 10.9** | 233.1*** | 25.2*** |
|  |  | D×N | 2.1ns | 18.2*** | 6.5* | 105.6** | 30.7** | 9.4** | 4.7* | 17.4*** | 110*** | 10.1** |
|  | IAA | ANOVA |  |  |  |  |  |  |  |  |  |  |
|  |  | Density(D) | 904.2*** | 213.5*** | 326.5*** | 125.6*** | 395.7*** | 789*** | 460.1*** | 371.4*** | 338.7*** | 820.5*** |
|  |  | N rate(N) | 58.8*** | 172.9*** | 283.5*** | 281.4*** | 346.5*** | 209.9*** | 313.3*** | 392*** | 148.7*** | 160.6*** |
|  |  | D×N | 4.2* | 10.2** | 11.4** | 20.8*** | 2.9ns | 6.3* | 15.4*** | 7.3* | 4.5*** | 14.8ns |
